# Human Mat2A Uses an Ordered Kinetic Mechanism and Is Stabilized but Not Regulated by Mat2B

**DOI:** 10.1021/acs.biochem.1c00672

**Published:** 2021-11-15

**Authors:** Jonathan Bailey, Holly Douglas, Laura Masino, Luiz Pedro Sorio de Carvalho, Argyrides Argyrou

**Affiliations:** † Mycobacterial Metabolism and Antibiotic Research Laboratory, The Francis Crick Institute, London NW1 1AT, United Kingdom; ‡ Structural Biology Scientific Technology Platform, The Francis Crick Institute, London NW1 1AT, United Kingdom; § Discovery Sciences, R&D, AstraZeneca, Cambridge CB4 0WG, United Kingdom

## Abstract

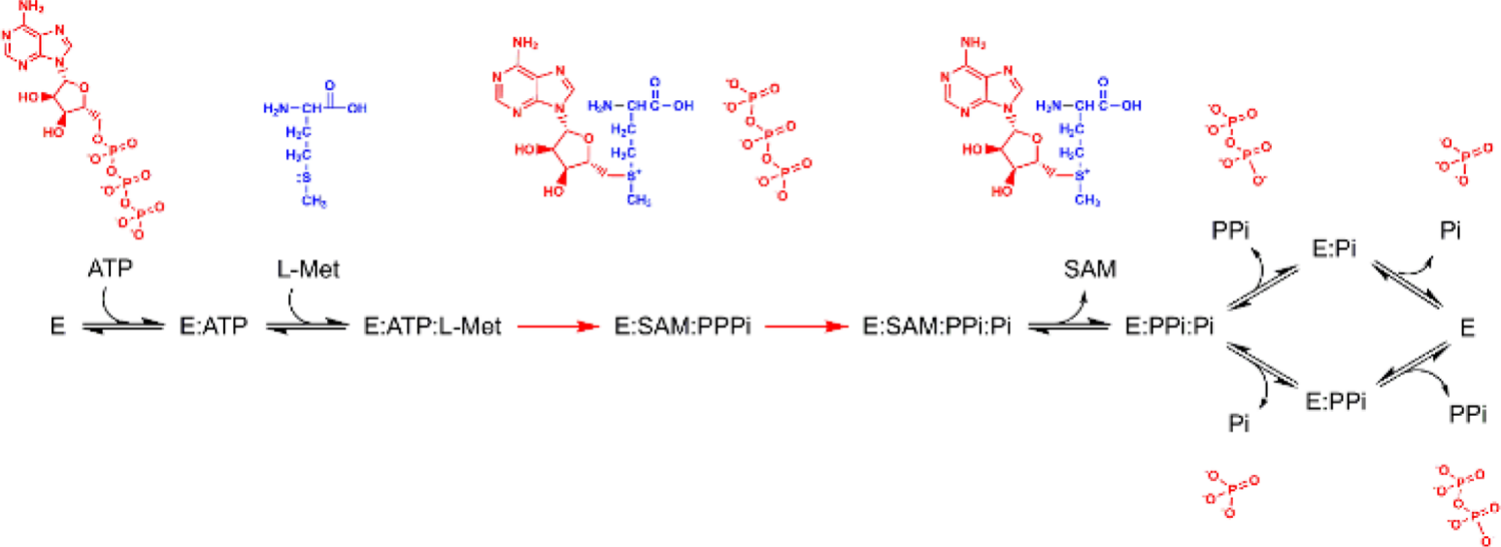

Methionine adenosyltransferase (MAT) catalyzes the adenosine 5′-triphosphate (ATP) and l-methionine (l-Met) dependent formation of *S*-adenosyl-l-methionine (SAM), the principal methyl donor of most biological transmethylation reactions. We carried out in-depth kinetic studies to further understand its mechanism and interaction with a potential regulator, Mat2B. The initial velocity pattern and results of product inhibition by SAM, phosphate, and pyrophosphate, and dead-end inhibition by the l-Met analog cycloleucine (l-cLeu) suggest that Mat2A follows a strictly ordered kinetic mechanism where ATP binds before l-Met and with SAM released prior to random release of phosphate and pyrophosphate. Isothermal titration calorimetry (ITC) showed binding of ATP to Mat2A with a *K*_d_ of 80 ± 30 μM, which is close to the *K*_m(ATP)_ of 50 ± 10 μM. In contrast, l-Met or l-cLeu showed no binding to Mat2A in the absence of ATP; however, binding to l-cLeu was observed in the presence of ATP. The ITC results are fully consistent with the product and dead-inhibition results obtained. We also carried out kinetic studies in the presence of the physiological regulator Mat2B. Under conditions where all Mat2A is found in complex with Mat2B, no significant change in the kinetic parameters was observed despite confirmation of a very high binding affinity of Mat2A to Mat2B (*K*_d_ of 6 ± 1 nM). Finally, we found that while Mat2A is unstable at low concentrations (<100 nM), rapidly losing activity at 37 °C, it retained full activity for at least 2 h when Mat2B was present at the known 2:1 Mat2A/Mat2B stoichiometry.

## Introduction

*S*-Adenosyl-l-methionine (SAM) is the principal methyl donor in biology and as such is an essential metabolite required for all forms of life. Almost all transmethylation reactions, including the methylation of DNA, RNA, histones, lipids, proteins, and secondary metabolites, utilize SAM as the methyl donor. SAM is a substrate for radical SAM superfamily (RSS) enzymes, which are responsible for performing a diverse array of vital radical reactions. RSS enzymes are involved in the synthesis of a large number of chemically distinct compounds, such as complex metal cofactors (e.g., HydE and HydG), organic cofactors (for instance, biotin and lipoic acid), and natural products (for example, the antibiotic nosiheptide) and usually involve the formation of a 5′-deoxyadenosyl radical intermediate.^1−4^ SAM is also involved in polyamine and glutathione biosynthesis and one-carbon metabolism via the folate cycle.^5−7^ Thus, alterations in the intracellular concentration of SAM might affect cell growth, cell death, cell differentiation, and a myriad of other processes.

SAM is synthesized by methionine adenosyltransferase (Mat; EC 2.1.5.6) using adenosine 5′-triphosphate (ATP) and l-methionine (l-Met) as substrates generating SAM, inorganic phosphate (P_i_), and pyrophosphate (PP_i_) as products.^8,9^ Humans possess two Mat isozymes, Mat1A and Mat2A, which are encoded by the *mat1a* and *mat2a* genes*,* respectively. These enzymes are highly similar, sharing 84% primary sequence identity and 93% sequence similarity, and have nearly identical structures (RMSD <1 Å).^10^ Despite their structural similarities, the two isozymes are differentially expressed.^6^ Mat1A is expressed exclusively in the liver where 85% of methylation reactions and the bulk of methionine metabolism occur. Mat2A is more widely expressed and is the main source of SAM outside of the liver, including in cancer cells.^6^ The focus of this study is the ubiquitously expressed and pharmacologically validated anti-cancer target Mat2A.

**Figure 1:**
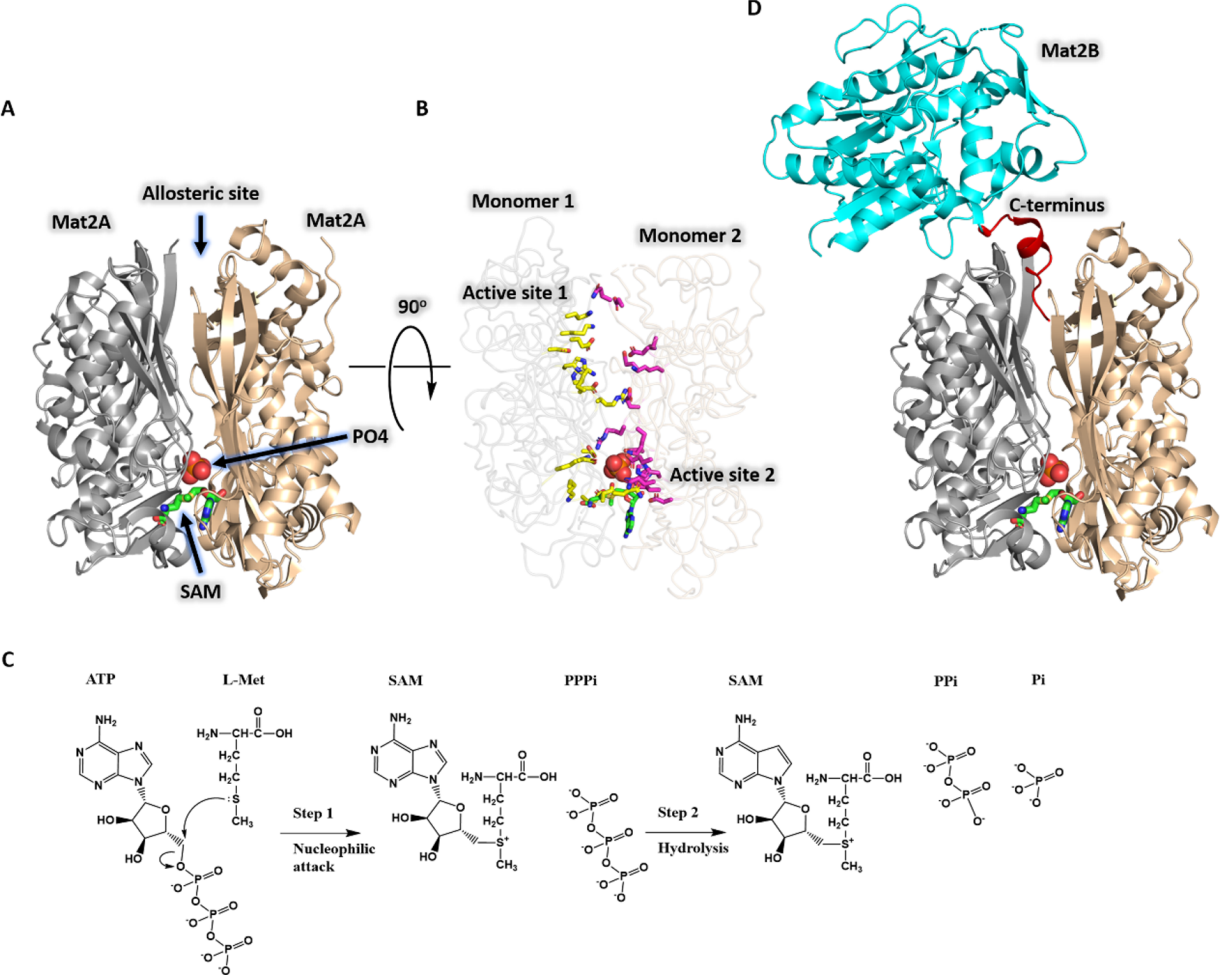
The structure and overall reaction catalyzed by human Mat2A. (A) The dimeric structure of Mat2A (cartoon representation) with the products SAM (stick representation, with green carbon atoms) and P_i_ (sphere representation, with orange phosphorus and red oxygen atoms) occupying one active site. Mat2A monomers are colored in gray and wheat. (B) As in panel A, viewed from the allosteric site (90° rotation) to show the two active sites of the Mat2A homodimer with one site empty and one site occupied by SAM and P_i_. Mat2A main chains are transparent to clearly show the active sites. Active site residues are shown with side chains in stick representation with yellow or pink carbon atoms. (C) As in panel A with Mat2B bound via its C-terminus to an allosteric site in the Mat2A dimer. Mat2B is colored in cyan and its C-terminus is red (PDB ID 4KTT).^13^ (D) The overall reaction catalyzed by Mat2A occurs via two distinct steps. The first step involves the sulfur atom of l-Met performing a nucleophilic attack on ATP, forming the product SAM and the intermediate tripolyphosphate (PPP_i_). The second step involves the hydrolysis of PPP_i_ that precedes the release of products SAM, PP_i_, and P_i_.

Mat2A is a functional homodimer with two symmetrical active sites (Figure 1A,B). The active sites are formed by residues contributed by both monomers, and therefore, dimerization is absolutely required for catalysis (Figure 1B).^10−15^ The Mat2A catalyzed reaction is proposed to occur in two steps, with an advanced S_N_2 transition state for SAM formation.^16^ Upon substrate binding, the first step of the reaction occurs when the sulfur atom of l-Met performs a nucleophilic attack on the 5′-carbon of ATP forming the product SAM and the intermediate tripolyphosphate (PPP_i_). The second step in the reaction is the hydrolysis of enzyme-bound PPP_i_, which occurs at the β-γ bond, generating P_i_ and PP_i_. Following PPP_i_ hydrolysis, the products SAM, PP_i_ and P_i_ are released (Figure 1C).^9,16−18^ Despite its importance for human health and disease, there are conflicting reports as to the steady-state kinetic mechanism of Mat2A. For example, sequential ordered kinetic mechanisms with ATP binding first or with l-Met binding first and sequential random binding of substrates have all been proposed, as well as proposals for ordered and random release of products.^12,16,19−21^ This confusion is likely due to experimental differences and potentially due to the presence of Mat2B bound to Mat2A in work carried out prior to recombinant protein expression using purified enzyme from human leukemia cells, which could affect the order of substrate binding and/or product release.

Animals possess Mat2B, a protein often described as a regulator of Mat2A activity.^10,13^ Humans possess two major splicing variants of the *mat2b* gene, which encode for Mat2B V1 and Mat2B V2.^22^ The amino acid sequences of Mat2B V1 (334 residues, MW 37.6 kDa) and Mat2B V2 (323 residues, MW 36.4 kDa) are identical except for the first 20 amino acids of the N-terminus (Figure S1), and both have been shown to interact with Mat2A with nanomolar affinity.^13,22^ Mat2B was first identified when it was co-purified with Mat2A from human leukemia cells and was soon found to be widely expressed in many of the same cells as Mat2A.^19,22,23^ Mat2B is not a homolog of Mat enzymes, sharing just 7% amino acid sequence identity, and has no known catalytic activity. A crystal structure of the Mat2A/Mat2B complex showed that monomeric Mat2B binds via its C-terminus to the Mat2A dimer, approximately 20 Å from the active site (Figure 1D).^13^ Isothermal titration calorimetry (ITC) assays confirmed the 2:1 Mat2A/Mat2B stoichiometry observed in the crystal structure of the complex and revealed a tight binding interaction, with a dissociation constant (*K*_d_) of approximately 4 nM.^10,13^ Mat2B is not believed to be expressed in the liver and therefore is not thought to be a physiological regulator of Mat1A. In addition, although Mat1A has been shown to bind Mat2B, the interaction is weaker with a *K*_d_ of 3 μM.^13^

Mat2A has been identified as a promising target for novel cancer therapies with the discovery that it is synthetically lethal with the enzyme methylthioadenosine phosphorylase (MTAP). MTAP is deleted in around 15% of cancers, and therefore, Mat2A inhibitors are attractive candidates for targeting cancers with MTAP deletion.^24,25^ Several potent inhibitors of Mat2A have recently been described (PF-9366, AG-270, compound 28).^10,24,26^ Interestingly, these inhibitors bind to the same site as Mat2B.^10,26,27^ AG-270 and compound 28 have been shown to inhibit cancer cell growth and proliferation of MTAP-deficient cancer cells and tumors. AG-270 has entered human clinical trials.^24,28^

The primary aim of this study was to determine the true steady-state kinetic mechanism of Mat2A using a combination of kinetics and binding experiments. Knowledge of the mechanism would assist with the design of assays, both enzymatic and binding assays, capable of identifying novel and improved inhibitors and understanding their mechanism of regulation by small molecule drug candidates and by the physiological regulator Mat2B. The second objective was to determine the effect of Mat2B on the kinetic constants of Mat2A to help understand how it regulates Mat2A activity. This is additionally important as Mat2B binds at the same allosteric site on Mat2A as all known Mat2A inhibitors, including compounds in human clinical trials. Therefore, in the cellular milieu, allosteric inhibitors could compete with Mat2B, decreasing their cellular potency and, ultimately, efficacy.

## Materials and Methods

### Materials

All chemicals were of analytical or reagent grade and were used without further purification unless otherwise stated. ATP(BioXtra), l-Met, SAM, TCEP, MgCl_2_, KCl, pyrophosphate, inorganic phosphate, formic acid, inorganic phosphatase from *Escherichia coli*, Benzonase Nuclease, and lysozyme from chicken egg white were purchased from Merck. Imidazole, IPTG, kanamycin, LB, and LC–MS grade methanol, acetonitrile, and H_2_O were from ThermoFisher. *E. coli* BL21 STAR (DE3) cells, NuPAGE MES SDS-PAGE running buffer, and NuPAGE 4–20% SDS-PAGE gels were from Invitrogen.

### Mat2A and Mat2B Constructs

The plasmid encoding human Mat2A was kindly provided by the Structural Genomics Consortium (SGC) Oxford (www.thesgc.org/structures/2p02). Briefly, the recombinant construct contained the full-length human Mat2A cDNA cloned into the pNIC28-Bsa4 vector (GenBank: EF198106.1). The expression construct was the full-length human Mat2A (UniProt P31153) with an additional 22 amino acid N-terminal sequence containing a His_6_ tag and tobacco etch virus protease (TEVp) cleavage site. The plasmid encoding human Mat2B (UniProt Q9NZL9) was provided by AstraZeneca. The expression construct was the full-length human Mat2B V2 (GenBank: AAH05218.1) with an additional 20 amino acid N-terminal sequence containing a His_6_ tag and TEVp cleavage site, cloned into a pET modified vector. DNA sequencing (Eurofins) confirmed the sequences.

### Expression and Purification of Mat2A and Mat2B

Identical protocols were used for expression of both Mat2A and Mat2B. All growth media were supplemented with 50 μg/mL kanamycin. *E. coli* BL21 STAR (DE3) were transformed with plasmids using the heat-shock method (42 °C for 45 s) and grown on Luria–Bertani (LB) agar plates for 16–18 h at 37 °C. Starter cultures were made by inoculating 100 mL of the LB broth with a single colony from LB agar plates. Starter cultures were grown for 20 h at 37 °C, 200 rpm. For protein expression, 5 mL of the starter culture was used to inoculate 500 mL of LB in 2 L flasks. Cells were grown at 37 °C, 200 rpm until an OD_600_ of approximately 0.6, and the temperature was reduced to 22 °C. Protein expression was induced with 0.5 mM IPTG. Expression was performed for 22 h at 20 °C, 200 rpm. Cells were pelleted by centrifugation at 4000*g* for 20 min at 4 °C and stored at −80 °C.

Cells were thawed at room temperature and suspended in 100 mL of buffer A (50 mM Tris/HCl pH 7.5, 100 mM KCl, 10 mM MgCl_2_, 1 mM TCEP (final pH 7.5)), and 2× Complete protease inhibitor tablets (Roche) were added. From this point, all samples were kept on ice or at 4 °C. Lysozyme from chicken egg white was added at a final concentration of approximately 1 mg/mL, and the samples were incubated for 1 h with agitation using a magnetic stirring bar. Cells were further lysed by probe sonication (amplitude 35%, on 12 s, off 59 s, 2 min total on time per cycle, 3–5 × cycles). Benzonase Nuclease (25 μL) was added to the lysed cells, and samples were incubated for 30 min with agitation using a magnetic stirring bar. Clarification was performed by centrifugation at 40,000*g*, 60 min. The clarified supernatant (approximately 100 mL volume) was transferred to a 150 mL superloop (GE Healthcare) and connected to the ÄKTA pure (GE Healthcare) chromatography system. Immobilized metal-ion affinity chromatography (IMAC) was performed using a 5 mL HisTrap HP (GE Healthcare) column. After loading, resin was washed with 50 mL of buffer A + 50 mM imidazole followed by elution using an imidazole gradient from 50 to 500 mM, achieved by increasing the ratio of buffer B to buffer A (buffer B = buffer A + 500 mM imidazole pH 8.0 (final pH 7.5)). *A*_280 nm_ peak fractions were collected, and the contents were analyzed by SDS-PAGE. Fractions containing the protein of interest (Mat2A or Mat2B) were pooled. If necessary, the pooled samples were diluted to approximately 2–3 mg/mL (≈50–70 μM) using buffer A. TEVp was added at a final concentration of 30 μg/mL (1 μM), transferred to a Spectra/Por 1 dialysis membrane (6–8 kDa MWCO) (Spectrum labs), and dialyzed against 1 L of buffer A for 18 h to remove imidazole and facilitate cleavage of the N-terminal tag. After TEVp cleavage, the uncleaved protein, TEVp, and N-terminal tag were removed by binding to Ni-NTA resin. PurKine His-tag Ni-NTA resin (2 mL; Abbkine) (pre-equilibrated with 20 mL of buffer A + 10 mM imidazole) was added to the sample, and binding was performed for 2 h. The sample was loaded onto a gravity column (Bio-Rad), and the flow-through, containing TEVp cleaved Mat2A or Mat2B, was collected. For size-exclusion chromatography (SEC), the TEVp cleaved protein was concentrated by centrifugation at 3000*g* using a Vivaspin-20 ultrafiltration unit (30,000 Da MWCO) (Sartorius) in 10 min intervals (sample homogenized by pipetting between intervals) to a volume of approximately 2 mL. Samples were loaded into a 5 mL loop on the ÄKTA pure (GE Healthcare) chromatography system, and SEC was performed using a HiLoad 16/600 Superdex 200 column (GE Healthcare) (Figure S2C,D). *A*_280 nm_ peak fractions were analyzed by SDS-PAGE, and samples containing the protein of interest, pooled and concentrated (as described above) to a final concentration of approximately 6–24 mg/mL (100–600 μM), were measured using a NanoDrop spectrophotometer (Mat2A ε_280 nm_ = 44,350 M^–1^ cm^–1^, MW = 43,748 Da; Mat2B ε_280 nm_ = 36,440 M^–1^ cm^–1^, MW 36683 Da). The pure protein was divided into 50 μL aliquots, flash frozen in liquid nitrogen, and stored at −80 °C. The purified proteins were analyzed by SDS-PAGE to assess purity (Figure S2A,B) and subjected to intact mass spectrometry to confirm identity and integrity. The molecular weights measured were within 0.1% of the expected molecular weight (based on primary amino acid sequence) (Figure S2E,F). The final yields were 17 mg/L Mat2A and 22 mg/L for Mat2B.

### Mat2A Enzyme Assays

Mat2A enzyme activity was monitored using two different assays under initial rate conditions at 22 °C in 50 mM Tris pH 7.5, 100 mM KCl, 10 mM MgCl_2_, and 1 mM TCEP buffer (buffer A, same as the protein purification buffer). In all cases, initial velocities were obtained from the linear regions of the reaction progress curves (<200 s, Figures S3C and S5**)**. In the majority of cases, substrate depletion was less than 10%; however, at the lowest l-Met concentrations, substrate depletion was unavoidably higher than 10% due to the low l-Met *K*_m_ but the progress curves were still largely linear. All assays were initiated by the addition of Mat2A except for the stability assays that were initiated with ATP.

#### Assay 1: MESG EnzChek Phosphate Assay

This assay is commercially available as a kit (ThermoFisher Cat. #E-6646), and it couples the phosphate product of the Mat2A reaction to phosphorolysis of 2-amino-6-mercapto-7-methylpurine ribonucleoside (MESG) catalyzed by purine nucleoside phosphorylase (PNP) to generate ribose-1-phosphate and the 2-amino-6-mercapto-7-methylpurine base, resulting in an increase in absorbance at 360 nm (Δε_360 nm_ = 11,000 M^–1^ cm^–1^).^29^ The assay concentrations of MESG and PNP were 200 μM and 1 U/mL, respectively, as recommended in the instructions provided by the manufacturer. *E. coli* inorganic pyrophosphatase (PP_i_ase) was included in all assay mixtures at a final concentration of 250 nM, with the only exception being the pyrophosphate product inhibition assays. PP_i_ase was added to convert the Mat2A product pyrophosphate into two molecules of inorganic phosphate, which increased the *A*_360 nm_ signal 3-fold (Figure S2D). Assays were performed in micro UV-cuvettes (BRAND), and absorbance was recorded using a UV-2550 spectrophotometer (SHIMADZU). The concentration of Mat2A was 250 nM. Initial rates were converted from Δ*A*_360_ s^–1^ to μM phosphate s^–1^ using a phosphate standard curve (Figure S3D) and dividing by three to account for the 3-fold boost in activity afforded by the action of PP_i_ase on the pyrophosphate product (Figure S3E). This method of converting change in absorbance to product formed is in excellent agreement with that obtained directly using the above change in extinction coefficient (Δε_360 nm_ = 11,000 M^–1^ cm^–1^).

#### Assay 2: Liquid Chromatography–Mass Spectrometry (LC–MS) Assay

The LC–MS assay monitors SAM directly and was developed to confirm the kinetic parameters that were obtained in the phosphate coupled assay and to carry out product inhibition studies by P_i_, which was not possible using the phosphate coupled assay. The amount of SAM produced was quantified using a standard curve (Figure S3F). Prior to LC–MS, 1:1 methanol/acetonitrile + 0.1% (*v*/*v*) formic acid was added to the samples. LC–MS was performed using an Agilent 1200 LC system fitted with a Cogent Diamond Hydride Type C silica column (2.1 × 150 mm, dead volume 315 μL). Solvent A was LC–MS grade H_2_O + 0.1% (*v*/*v*) formic acid. Solvent B was acetonitrile + 0.1% (*v*/*v*) formic acid. The gradient started at 85% B dropping to 5% B over 14 min. Flow rate was 0.4 mL/min. Mass spectrometry was performed using an Agilent Accurate Mass 6230 TOF apparatus in positive-ion mode. The doubly charged SAM ion was detected at an *m/z* of 200.0759.

### Isothermal Titration Calorimetry (ITC) Binding Assays

Prior to performing ITC, proteins (approximately 1 mL volume) were dialyzed using D-Tube Dialyzers (Maxi 100–3000 μL, MWCO 6–8 kDa) (Novagen) against 1 L of buffer A for 2–4 h at 4 °C with agitation using a magnetic stirring bar. Ligands (ATP, l-Met, and l-cLeu) were reconstituted using the same dialysis buffer after dialysis had been completed. Titrations were performed at 20 °C. Protein concentrations were determined by *A*_280_ using a UV-2550 spectrophotometer. The protein and ligand concentrations that were used are indicated in the legends of Figures 5 and 6. ITC measurements were performed on a MicroCal PEAQ-ITC calorimeter (Malvern Panalytical). Data were analyzed using the MicroCal PEAQ-ITC analysis software supplied by the manufacturer using nonlinear regression with the One Set of Sites model. For each experiment, the heat produced or consumed associated with ligand dilution was measured and subtracted from the raw data.

### Data Analysis

All initial rate data were fitted using either SigmaPlot 14.0 or GraphPad Prism 9.0.0. Initial velocity patterns for Mat2A were determined by measuring initial rates at varying concentrations of ATP and multiple fixed concentrations of l-Met. Steady-state kinetic parameters (*k*_cat_, *K*_m(ATP)_, *K*_m_(l-Met), and *K*_i(ATP)_) were calculated from these data by fitting the data to the rate equation for ternary complex mechanism, eq 1.
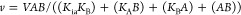
1

where *V* is the maximal velocity; *A* and *B* are the concentrations of ATP and l-Met, respectively; *K*_A_ and *K*_B_ are the Michaelis constants for ATP and l-Met, respectively; and *K*_ia_ is the dissociation constant of the enzyme–ATP complex.

Product and dead-end inhibition data showing competitive, noncompetitive, or uncompetitive patterns were fitted to eqs 2–4, respectively.
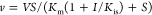
2
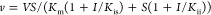
3
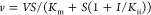
4where *I* is the inhibitor concentration; *S* is the concentration of the variable substrate; *K*_m_ is the Michaelis constant for the variable substrate; and *K*_is_ and *K*_ii_ are the slope and intercept inhibition constants, respectively.

Saturation kinetics data were fitted to eq 5.
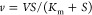
5

Stability assay data were fitted to eq 6.
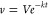
6where *V* is the enzyme activity at time zero, *k* is the first-order rate constant describing the loss of activity, and *t* is time.

## Results

### Expression, Purification, and Biophysical Characterization of Mat2A and Mat2B

Recombinant Mat2A and Mat2B were expressed separately using *E. coli* BL21(DE3) cells and purified by Ni-NTA affinity chromatography followed by size-exclusion chromatography (SEC) to homogeneity (Figure S2A,B). The SEC chromatograms show single, almost symmetrical peaks suggesting that both proteins purify in a homogeneous state (Figure S2C,D). The molecular weight of the purified proteins was determined by intact mass spectrometry and was within 0.1% of the predicted molecular weight based on the primary amino acid sequences, thus confirming the identity and integrity of the purified proteins (Figure S2E,F).

### Mat2A Steady-State Kinetics

**Figure 2:**
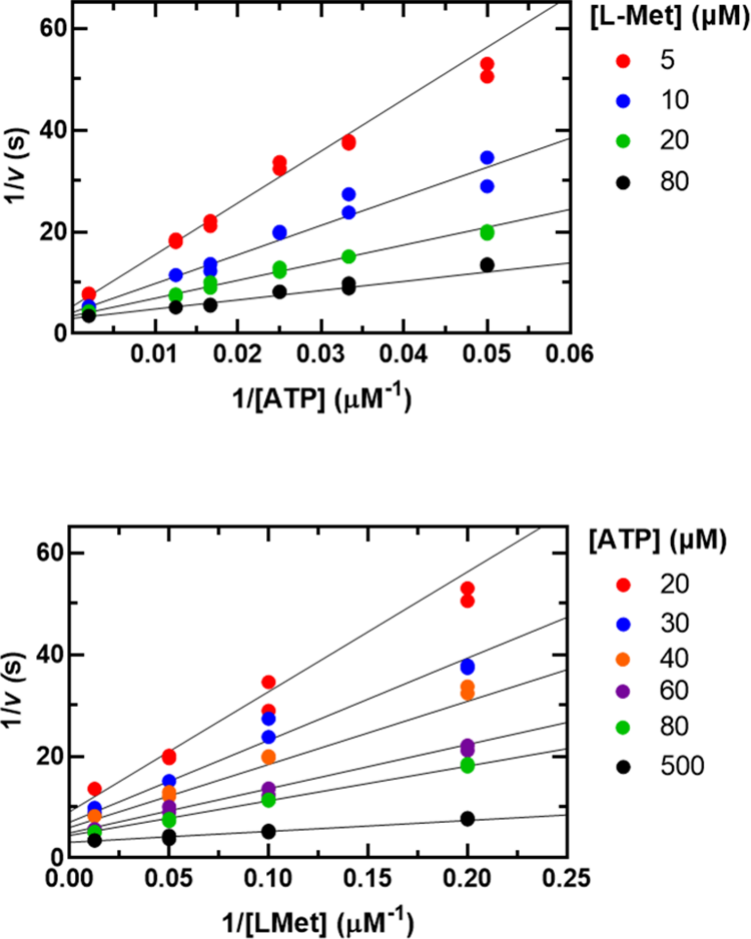
Initial velocity pattern for Mat2A using assay 1. The ATP concentration was varied (20–500 μM) at several fixed l-Met concentrations (5–80 μM). Symbols show experimental data, and solid lines represent a global fit of all data points to eq 1. Please note that the top and bottom graphs are representations of the same data, the difference being the substrate that is plotted on the *x* axis and the substrate that is held fixed at several levels as indicated.

To determine the steady-state kinetic constants (*k*_cat_ and substrate *K*_m_ values, Table 1) and to obtain information regarding the kinetic mechanism (ternary complex or ping-pong), we used a phosphate detection coupled assay (see Materials and Methods), varied one substrate at several fixed levels of the co-substrate, and fitted all the initial velocity data globally to eq 1. We ensured that the coupling enzyme system was not rate-limiting by demonstrating that Mat2A activity was linear with Mat2A concentration (Figure S3A). A double reciprocal plot of the initial velocity data showed a pattern of intersecting lines suggesting that Mat2A uses a sequential kinetic mechanism (Figure 2) where both substrates are required to bind to the enzyme before chemistry can occur.^30^ The kinetic constants were also determined using a direct LC–MS SAM detection assay where initial rates were determined at a range of concentrations of one substrate and saturating concentrations of the second substrate (Figure S3B and Table 1). The kinetic constants were in good agreement between the phosphate detection coupled assay and the direct LC–MS SAM detection assay and remained the same when assays were performed at low (30 nM) or high (250 nM) concentrations of Mat2A.

**Table 1:** Steady-State Kinetic Constants of Human Mat2A^a^

	Mat2A only^b^^,^^c^	Mat2A only^d^^,^^e^	Mat2A + Mat2B^b^^,^^c^
*k*_cat_ (s^–1^)	0.34 ± 0.06	0.34 ± 0.02	0.32 ± 0.03
*K*_m(ATP)_ (μM)	50 ± 10	55 ± 10	50 ± 5
*K*_m_(l-Met) (μM)	5 ± 2	8 ± 2	4 ± 1
*K*_i(ATP)_ (μM)	349 ± 66	ND	183 ± 38

a At pH 7.5 and 22 °C.

b Coupled phosphate detection assay.

c Values are averages from five independent experiments, and errors are the standard deviation.

d LC–MS SAM detection assay.

e Values are from a single experiment with three replicates, and errors are the standard error. ND = not determined.

### Product Inhibition

**Figure 3:**
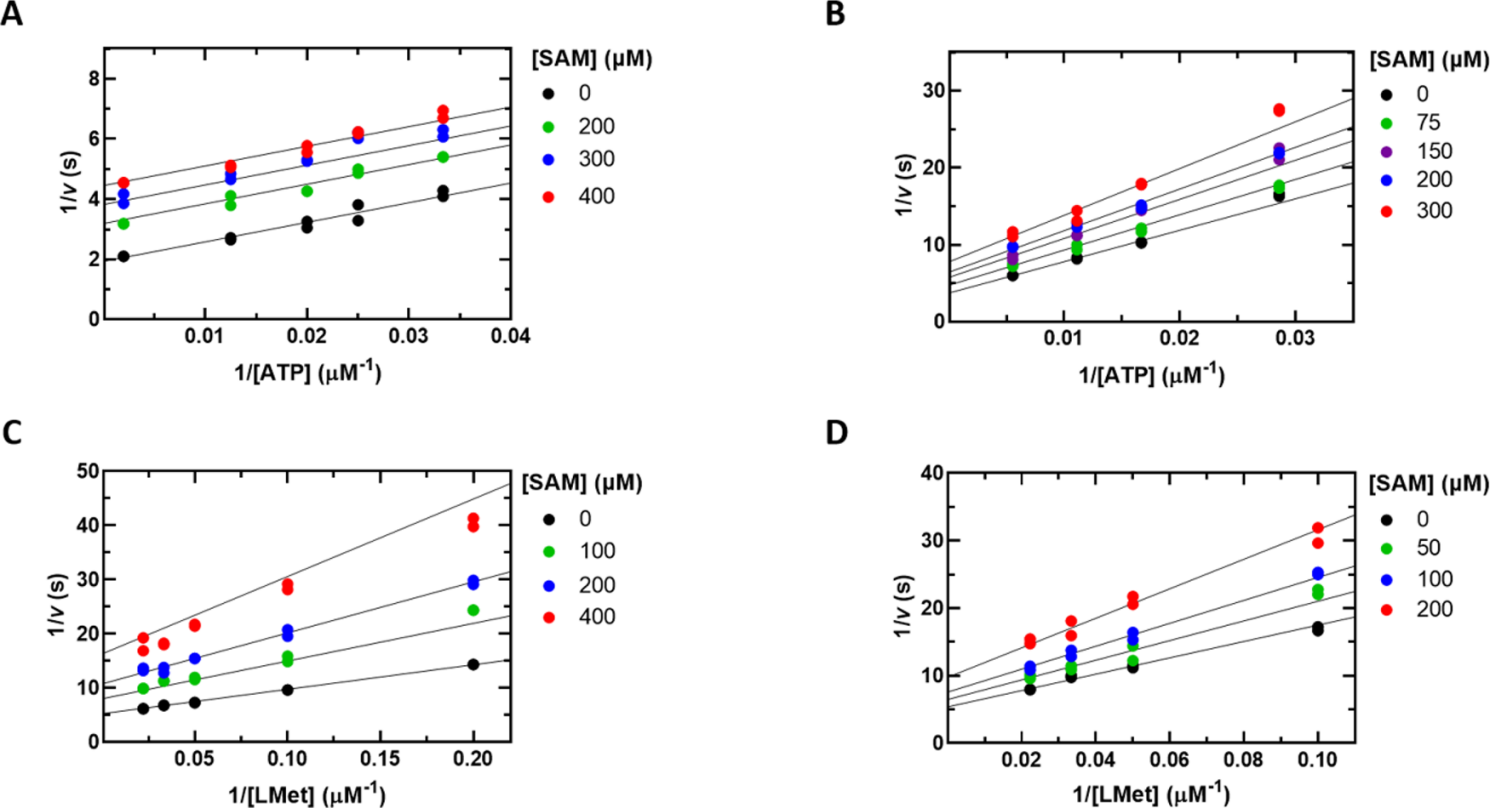
Product inhibition patterns by SAM using assay 1. (A) ATP was varied (30–500 μM) at a fixed saturating concentration of l-Met (600 μM) and at the indicated concentrations of SAM. (B) l-Met was varied (5–45 μM) at a fixed saturating concentration of ATP (500 μM) and at the indicated concentrations of SAM. (C) ATP was varied (20–180 μM) at a fixed nonsaturating concentration of l-Met (20 μM) and the indicated concentrations of SAM. (D) l-Met was varied (10–45 μM) at a fixed nonsaturating concentration of ATP (70 μM) and the indicated concentrations of SAM. Symbols show experimental data, and solid lines show fits of all data points to eq 4 in panel A and eq 3 in panels B, C, and D.

To investigate the order of substrate binding and release of products, product inhibition experiments were performed with the products SAM, PP_i_, and P_i_. At saturating l-Met, inhibition by SAM was uncompetitive versus ATP with a *K*_ii_ of 230 ± 50 μM (Figure 3A), whereas at nonsaturating l-Met, SAM inhibition was noncompetitive with a *K*_ii_ = 280 ± 30 μM and *K*_is_ = 620 ± 130 μM (Figure 3B). SAM inhibition versus l-Met was noncompetitive at both saturating and nonsaturating concentrations of ATP with inhibition constants of *K*_ii_ = *K*_is_ = 190 ± 10 μM and *K*_ii_ = *K*_is_ = 250 ± 10 μM, respectively (Figure 3C,D). Inhibition by PP_i_ and P_i_ was noncompetitive against both substrates (Figure S4). The results of these studies are summarized in Table 2.

**Table 2:** Product Inhibition Patterns and Inhibition Constants^a^

variable substrate	product	type of inhibition	inhibition constant (μM)
ATP	SAM	U^b^	*K*_ii_ = 230 ± 50^b^^*,*^^d^
		NC^c^	*K*_ii_ = 275 ± 30^c^
			*K*_is_ = 620 ± 130^c^
ATP	pyrophosphate	NC^b^	*K*_ii_ = 800 ± 100^b^^*,*^^e^
			*K*_is_ = 210 ± 10^b^^*,*^^e^
ATP	phosphate	NC^b^	*K*_ii_ = 5740 ± 760^b^
			*K*_is_ = 1290 ± 170^b^
l-Met	SAM	NC^b^	*K*_ii_ = *K*_is_ = 190 ± 10^b^
		NC^c^	*K*_ii_ = *K*_is_ = 250 ± 10^c^
l-Met	pyrophosphate	NC^b^	*K*_ii_ = 660 ± 40^b^
			*K*_is_ = 180 ± 20^b^
l-Met	phosphate	NC^b^	*K*_ii_ = 4970 ± 770^b^
			*K*_is_ = 1580 ± 360^b^

a At pH 7.5 and 22 °C.

b Saturating concentrations of the co-substrate: 0.6 mM l-Met, 0.5 or 1 mM ATP.

c Nonsaturating concentrations of the co-substrate.

d Average of six independent experiments; error shows standard deviation.

e Average of two independent experiments; error shows standard deviation: 0.02 mM l-Met, 0.07 mM ATP. U indicates uncompetitive inhibition and NC indicates noncompetitive inhibition.

### Dead-End Inhibition

**Figure 4:**
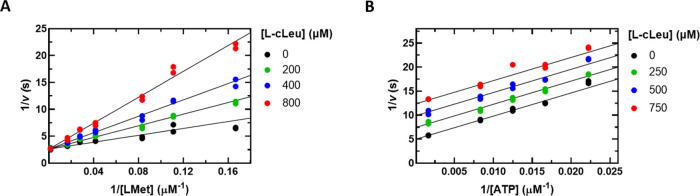
Inhibition patterns by the methionine analogue l-cLeu using assay 1. (A) l-Met was varied (6–600 μM) at a fixed saturating concentration of ATP (1 mM) and at the indicated concentrations of l-cLeu. (B) ATP was varied (45–500 μM) at a fixed nonsaturating l-Met concentration (20 μM) and at the indicated concentrations of l-cLeu. Symbols show experimental data, and solid lines show simultaneous fits of all data points to eq 2 in panel A and eq 4 in panel B.

To gain further insight into the order of substrate binding, inhibition experiments were performed using the l-Met analogue, l-cycloleucine (l-cLeu). l-cLeu is not a substrate for Mat2A but was found to fully inhibit the enzyme. l-cLeu displays competitive inhibition versus l-Met at saturating and nonsaturating ATP, with *K*_is_ values of 290 ± 30 and 160 ± 20 μM, respectively (Figure 4A and Table 3). No inhibition was observed when ATP was varied and l-Met concentrations were saturating (600 μM), consistent with l-cLeu being a competitive inhibitor versus l-Met. At nonsaturating l-Met concentration (20 μM), l-cLeu inhibited Mat2A uncompetitively versus ATP, with a *K*_ii_ of 510 ± 20 μM (Figure 4B and Table 3). The results are tabulated in Table 3.

**Table 3:** Inhibition Patterns and Constants by the l-Met Analogue l-cLeu^a^

variable substrate	fixed substrate	inhibition pattern	inhibition constant (μM)
l-Met	saturating ATP	C	*K*_is_ = 290 ± 30
l-Met	nonsaturating ATP	C	*K*_is_ = 160 ± 20
ATP	saturating l-Met	NI	ND
ATP	nonsaturating l-Met	U	*K*_ii_ = 510 ± 20

a At pH 7.5 and 22 °C. Saturating ATP = 1 mM, Nonsaturating ATP = 0.07 mM, saturating l-Met = 0.6 mM, and nonsaturating l-Met = 0.02 mM. C indicates competitive inhibition, NI indicates no inhibition, and U indicates uncompetitive inhibition.

### Isothermal Titration Calorimetry Binding Experiments

**Figure 5:**
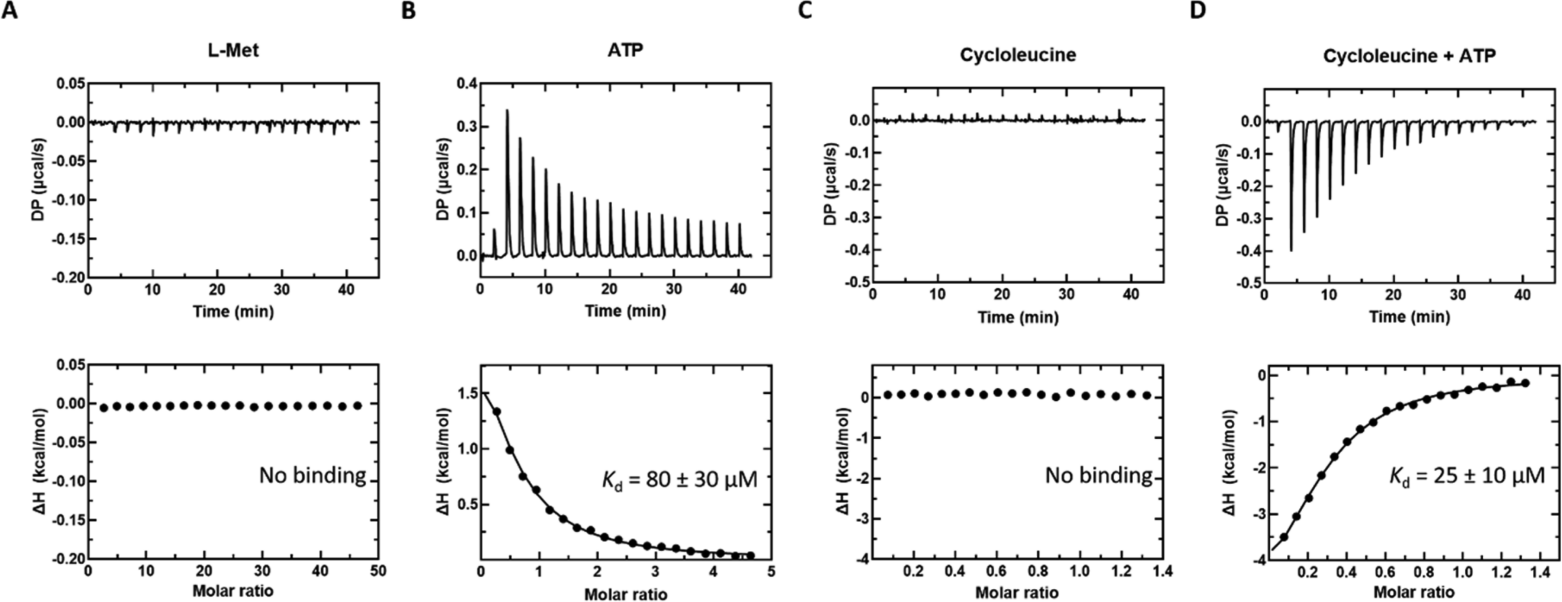
ITC thermograms of l-Met, ATP, and l-cLeu (± ATP) titrated into Mat2A. (A) l-Met (20 mM) titrated into Mat2A (87 μM). (B) ATP (2 mM) titrated into Mat2A (87 μM). (C) l-cLeu (2 mM) titrated into Mat2A (205 μM). (D) l-cLeu (2 mM) + ATP (2 mM) titrated into Mat2A (205 μM) + ATP (2 mM). All titrations performed at 20 °C. Unprocessed thermograms (top panels) and the binding isotherm from the integrated thermogram fit to the One Set of Sites model using the MicroCal PEAQ-ITC analysis software (bottom panels). Circles indicate the integrated heat, and the curve represents the best fit. Data shown are representative of four independent experiments.

Next, we sought to analyze substrate binding directly using isothermal titration calorimetry (ITC). Binding assays were carried out by titrating ATP, l-Met, l-cLeu, or l-cLeu + ATP into Mat2A in the absence of the co-substrate to avoid turnover. At 20 °C, binding of ATP to Mat2A is endothermic with a *K*_d(ATP)_ of 80 ± 30 μM. The measured *K*_d(ATP)_ agrees well with the *K*_m(ATP)_ of 50 ± 10 μM. No significant change in heat was observed when l-Met was added to Mat2A, even at very high l-Met concentrations, despite a *K*_m_(l-Met) of 5 ± 2 μM. Similarly, no significant change in heat was observed when l-cLeu was added to Mat2A. However, in the presence of 2 mM ATP, binding to l-cLeu was observed (Figure 5).

### Mat2B Binding to Mat2A

**Figure 6:**
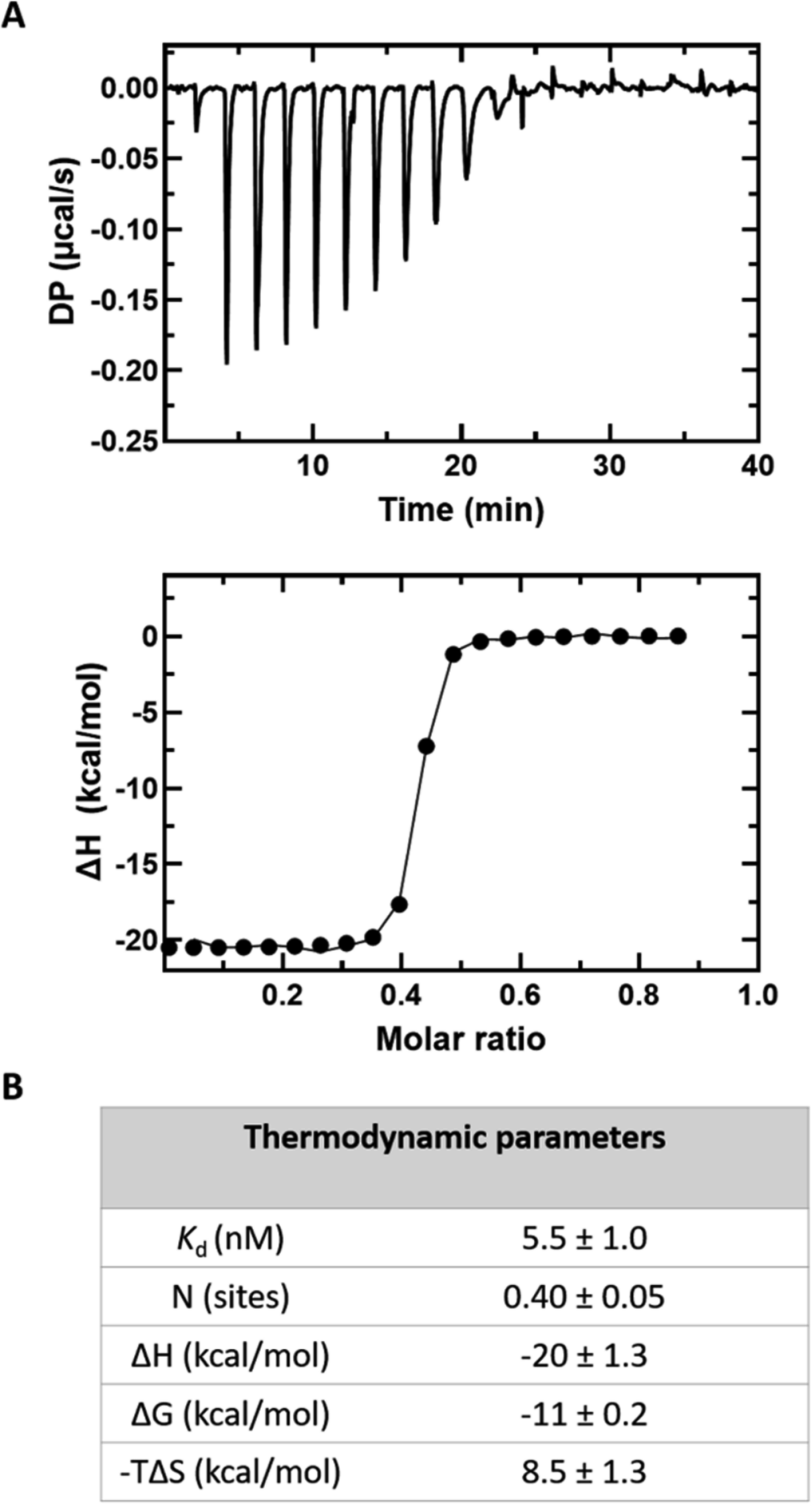
Mat2A binding to Mat2B in solution. (A) Mat2B (50 μM) titrated into Mat2A (14 μM). Raw thermogram (top panel) and the binding isotherm from the integrated thermogram fit using the One Set of Sites model in the MicroCal PEAQ-ITC analysis software (bottom panel). Circles indicate the integrated heat, and the curve represents the best fit. (B) Thermodynamic parameters. Reported values are averages from six independent titrations, and the error is the standard deviation.

Both major splicing variants of Mat2B (V1 and V2) are reported to bind tightly to Mat2A in solution with low nM affinity and a binding stoichiometry of 2:1 (Mat2A/Mat2B).^10,13^ To confirm these findings using our protein preparations, we carried out ITC experiments using Mat2B V2 (henceforth referred to as Mat2B). Mat2A was found to bind tightly to Mat2B with a *K*_d_ = 6 ± 1 nM and a stoichiometry 2:1 (Mat2A/Mat2B, *N* = 0.42 ± 0.02) (Figure 6), consistent with previous reports.^10,13^

### Steady-State Kinetics and Product Inhibition of the Mat2A/Mat2B Complex

Having established the formation of a high-affinity Mat2A:Mat2B complex under our experimental conditions, we sought to determine the effect of Mat2B binding on Mat2A activity. Initially, we kept Mat2A at 300 nM and the substrates at concentrations near their *K*_m_ values and varied the concentration of Mat2B from 0.075 to 15 μM. No inhibition or activation of Mat2A activity by Mat2B was observed in either the phosphate detection coupled assay or the LC–MS SAM detection assay (Figures S5 and S6). When the same types of experiments were performed using a published Mat2A inhibitor (compound 28) that binds at the same allosteric site and with an affinity similar to Mat2B (Mat2B *K*_d_ = 6 ± 1 nM, compound 28 *K*_d_ = 12 ± 2 nM), inhibition was observed (Figure S7), consistent with the recent literature.^26^

**Figure 7:**
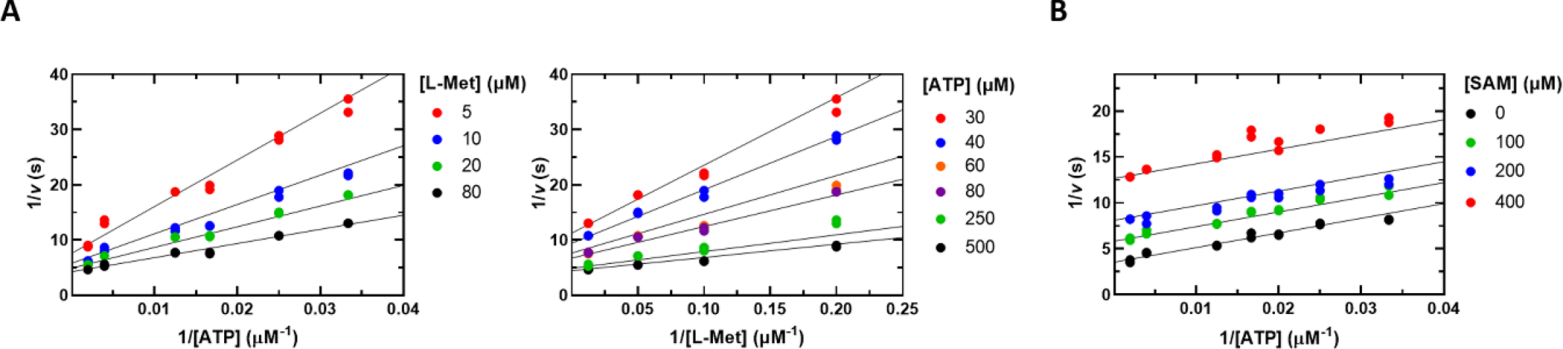
(A) Initial velocity pattern of the Mat2A/Mat2B complex using assay 1. ATP was varied (30–500 μM) at several fixed l-Met concentrations (5–80 μM). Please note that the left and right graphs are representations of the same data, the difference being the substrate that is plotted on the *x* axis and the substrate that is held fixed at several levels as indicated. (B) SAM product inhibition of the Mat2A/Mat2B complex. ATP was varied (30–500 μM) at a fixed saturating concentration of l-Met (600 μM) and at the indicated concentrations of SAM. Symbols show experimental data, and solid lines show simultaneous fits of all data points to eq 1 in panel A and eq 4 in panel B.

We next varied the ATP concentration at several fixed levels of l-Met using the Mat2A/Mat2B complex (250 nM Mat2A and 600 nM Mat2B) and fitted the data to eq 1 to determine the steady-state kinetic constants (Figure 7A and Table 1). The magnitude of the kinetic constants obtained with the Mat2A/Mat2B complex was very similar to the values obtained with Mat2A alone. Therefore, under these conditions, binding of Mat2B to Mat2A has no significant effect on the *k*_cat_ or *K*_m_ values. These results do not support previous conclusions that Mat2B is an inhibitor or an activator of Mat2A activity (see below and Discussion).^10,13,31−33^

The effects of Mat2B on SAM product inhibition were investigated. Inhibition by SAM remained uncompetitive in the presence of saturating Mat2B with a *K*_ii(SAM)_ of 130 ± 40 μM (Figure 7B), which is similar to the value of 230 ± 50 μM in the absence of Mat2B (Table 3). The physiological relevance of this small difference, if any, is unclear.

### Stability Assays

**Figure 8:**
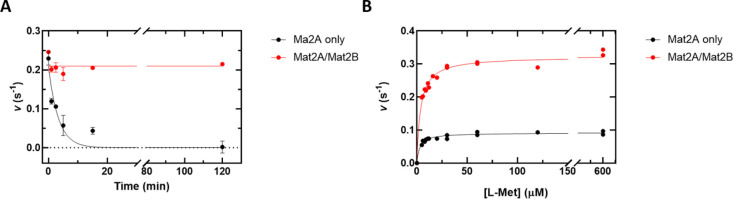
Mat2A activity is stabilized by Mat2B. (A) Mat2A activity remaining after preincubation of 60 nM Mat2A at 37 °C for 0, 1, 2.5, 5, 15, and 120 min in the absence and presence of 30 nM Mat2B. ATP concentration is 1.25 mM, and l-Met concentration is 200 μM. (B) Dependence of rate on l-Met concentration using 60 nM Mat2A or 60 nM Mat2A/30 nM Mat2B complex preincubated at 37 °C for 15 min. l-Met was varied (5–600 μM), and ATP is fixed at 1.25 mM. Symbols show experimental data, and the solid lines represent the best fits to eq 6 in panel A and eq 5 in panel B.

Lastly, we investigated the stability of Mat2A activity at more physiological concentrations in the absence and presence of Mat2B. When 60 nM Mat2A was preincubated in the assay buffer at 37 °C in the absence of substrates, time-dependent loss of enzyme activity was observed resulting in a 50% loss of activity in 2.3 min (Figure 8A). In the presence of 30 nM Mat2B during the preincubation period, however, the enzyme retained full activity for at least 120 min (Figure 8A)**.** We also measured the *k*_cat_ and *K*_m_(l-Met) values after 60 nM Mat2A was preincubated at 37 °C for 15 min in the absence and presence of 30 nM Mat2B. There was no significant difference in the *K*_m_(l-Met) in the presence or absence of Mat2B. However, in the absence of Mat2B, the *k*_cat_ was almost 4-fold lower than when Mat2B was included in the preincubation (Figure 8B and Table 4). The same effect was observed when activity was measured using the LC–MS SAM detection assay (Figure S8). We conclude that while Mat2A is unstable at low concentrations in the absence of substrates, the Mat2A/Mat2B complex is significantly more stable for at least 120 min. These results should not be misinterpreted as activation of Mat2A enzyme activity by Mat2B.^13,31^

**Table 4:** Mat2A ± Mat2B Kinetic Constants after Preincubation for 15 min at 37 °C^a^

kinetic constant	Mat2A only	Mat2A + Mat2B
*K*_m_(l-Met) (μM)	3.5 ± 1.3^b^	3.9 ± 0.1^b^
*k*_cat_ (s^–1^)	0.09 ± 0.005^b^	0.32 ± 0.006^b^

a At pH 7.5 and 22 °C.

b Values are average of three independent experiments; error is the standard deviation.

## Discussion

Human Mat2A is a pharmacologically validated target for the development of novel anti-cancer therapeutics for patients with MTAP-deleted cancers, which account for 15% of all cancers.^24,25^ There are conflicting reports describing Mat2A following random or ordered kinetic mechanisms.^12,20,21^ In addition, different reports have described Mat2B as an inhibitor or an activator of Mat2A.^10,13,31−33^ In this work, we present comprehensive steady-state kinetics and binding results that allowed us to determine the steady-state mechanism of human Mat2A and the effect of Mat2B.

Initial velocity experiments were performed at variable concentrations of ATP and several fixed concentrations of l-Met. Double reciprocal plots of the initial velocity data show a pattern of intersecting lines, diagnostic of a sequential mechanism where both substrates have to bind to the enzyme before catalysis can occur (Figure 2). In general, the steady-state kinetic parameters that we obtained are in good agreement with literature values; the substrate *K*_m_ values that we obtained (5 μM for l-Met and 50 μM for ATP) are consistent with previously reported values, which range from 3 to 50 μM for l-Met and 30 to 130 μM for ATP (Table 1).^10,13,19,21^ Of note, Mat2A appears relatively slow (*k*_cat_ = 0.34 s^–1^) to maintain the metabolic supply of SAM; however, the *k*_cat_ agrees well with values reported by other groups using different assay methods (0.27 and 0.35 s^–1^)^10,21^ and is remarkably within 2-fold^1^ to that reported using native Mat2A/Mat2B purified from human lymphocytes.^19,20^ The relatively slow turnover number might be more significant in rapidly dividing cells, such as cancer cells. It would be important to measure the absolute concentration of Mat2A (and Mat2B) in different cell types under different physiological conditions to determine if the *in vitro* rate of Mat2A is fast enough to maintain the metabolic supply of SAM. Recently, the RNA binding protein and methyltransferase METTL16 have been implicated in regulating cellular Mat2A concentrations in response to the levels of SAM.^34−36^ When SAM concentrations are high, METTL16, which utilizes SAM as a substrate, binds and methylates Mat2A mRNA resulting in intron retention and degradation, which cause a reduction in the cellular concentrations of the Mat2A protein and SAM.^37^ When cellular SAM concentrations are low, the methylation of Mat2A mRNA by METTL16 is reduced, resulting in increased levels of Mat2A mRNA being translated into the Mat2A protein and an increase in the cellular SAM concentration.^34−36^ Therefore, METTL16 acts as a SAM sensor that directly regulates the amount of Mat2A enzyme in the cell in response to cellular SAM concentrations. Another possibility is that the *in vivo* activity of Mat2A is greatly enhanced by forming complexes with other proteins, macromolecules, or metabolites, which is discussed in more detail below.^21^

**Scheme 1:**
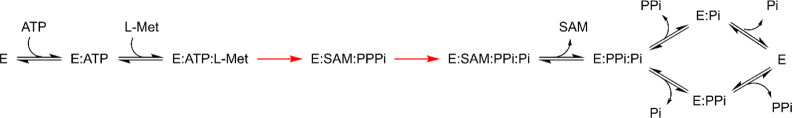
The Steady-State Mechanism of Human Mat2A Determined by Initial Velocity Patterns and Binding Assays Red arrows denote the chemical steps.

The most informative inhibition patterns are the competitive and uncompetitive patterns. When these are observed, there usually is a preferred order of substrate binding and release of products. For Mat2A, product inhibition by SAM was noncompetitive versus ATP at nonsaturating levels of l-Met but uncompetitive at saturating levels of l-Met (Figure 3). These results are diagnostic of an ordered steady-state kinetic mechanism in which ATP is the first substrate to bind and SAM is the first product released. Competitive product inhibition patterns allow one to identify the last product to be released and the first substrate to bind. Inhibition by the other products of the Mat2A reaction, PP_i_ and P_i_, was noncompetitive against ATP and l-Met at saturating concentrations of the co-substrate (Figure S4). The lack of an observed competitive product inhibition pattern for Mat2A suggests that the release of products PP_i_ and P_i_ is not strictly ordered. If product release were strictly ordered, inhibition by the final product released would be competitive against the first substrate to bind, as both bind to the same form of Mat2A (free enzyme).^30^ Dead-end inhibition experiments with the l-Met analogue l-cLeu showed competitive inhibition versus l-Met and uncompetitive inhibition versus ATP, suggesting that substrate binding is ordered with ATP binding first followed by l-Met (Figure 4). If substrate binding was random, inhibition by l-cLeu would be noncompetitive versus ATP.^30,38,39^ This conclusion is supported in the literature by kinetic isotope effect experiments that measured large and negligible forward commitment factors for ATP and l-Met, respectively, suggesting that ATP binds first to Mat2A.^16^ Taken together, the results of steady-state inhibition kinetics point to an ordered steady-state mechanism, with ATP binding first followed by l-Met. Product release is partially ordered, with SAM released first followed by a random release of products PP_i_ and P_i_ (Scheme 1).

The equilibrium binding results also support the proposed ordered steady-state mechanism. Binding of ATP to Mat2A was observed in the absence of l-Met consistent with the proposal that ATP binds first (Figure 5B). In contrast, binding of l-Met or l-cLeu to Mat2A was not detected in the absence of ATP (Figure 5A,C); however, binding to l-cLeu was detected in the presence of ATP (Figure 5D). These results suggest that the l-Met binding site is either disordered or inaccessible prior to ATP binding.

This study also sought to address the contradictory reported effects that Mat2B binding has on Mat2A activity. The role of Mat2B as a regulator of Mat2A activity arose in part from studies using crude extracts prepared from superantigen staphylococcal enterotoxin B (SEB)-stimulated human T lymphocytes, where Mat2B expression was suppressed, and mitogen phytohemagglutinin (PHA)-stimulated cells expressing both Mat2A and Mat2B. It was found that the *K*_m_ for l-Met was around 2- to 3-fold higher and *V_max_* was around 2-fold lower in SEB-stimulated than in PHA-stimulated cells. A 1.5-fold increase in potency of inhibition by the product SAM was also reported in cells expressing Mat2B.^33^ It was postulated that Mat2B is an inhibitory regulator of Mat2A activity that works by increasing the affinity for the substrate l-Met by lowering the *K*_m_, reducing the *V*_max_, and increasing the potency of product inhibition by SAM, the net effect being that Mat2B binding inhibits Mat2A activity causing a decrease in the cellular SAM concentration.

More recently, it has been reported that SAM concentrations are around 2-fold higher in H520 cells when Mat2B expression was knocked down.^10^ However, elsewhere, it has been reported that in experiments using crude extracts prepared from COS-1 cells, expressing Mat2B resulted in a 2-fold increase in native Mat2A specific activity and had no effect on the *K*_m_ for l-Met.^32^ A 2-fold increase in the potency of product inhibition by SAM was also reported.^32^ Without detailed and rigorous mechanistic studies, it is not possible to determine if the observed relatively small differences in Mat2A activity and cellular SAM concentrations in crude cell extracts prepared from various cell lines are truly due to changes in Mat2A catalytic activity effected by Mat2B binding or if it could be explained by other cellular processes resulting from knocking down Mat2B expression or increased degradation.

Contradictory reports also exist as to the effect of Mat2B on Mat2A activity *in vitro.* It has been reported that addition of Mat2B decreased the *K*_m_ for l-Met from 14 to 2.5 μM and that the potency of product inhibition by SAM increased 2-fold, reducing the IC_50_ from around 300 to 150 μM.^10^ The same study reported that Mat2B inhibited Mat2A in a dose–response manner, saturating at 50% inhibition.^10^ Other groups have reported that Mat2B has no effect on the *K*_m_ for l-Met but that it activates Mat2A activity 3- to 4-fold.^13,31^ These studies incorporated a preincubation step, in which Mat2A was incubated for 15 min at 37 °C prior to assays being performed. Under these conditions, Mat2A is unstable and quickly loses activity (50% in 2.3 min). In the presence of Mat2B, however, the enzyme is stable and loses no activity for at least 120 min at 37 °C (Figure 8). Without prior knowledge that Mat2A is unstable under these conditions, the stabilizing effect of Mat2B was probably misinterpreted as activation of Mat2A.^13,31^ Therefore, Mat2B has been reported to be an inhibitor in some studies but an activator of Mat2A activity in other studies. The reported effects are relatively small and could be explained by differences in experimental conditions. The most consistently reported effect is an approximately 2-fold increase in the potency of product inhibition by SAM. It is unclear if this relatively minor change in potency is physiologically relevant. For example, in human lymphocytes, cellular SAM concentrations have been reported as 20 μM in resting lymphocytes and 100 μM in activated lymphocytes.^32^

Here, we present evidence that Mat2B has no effect on Mat2A catalytic activity using purified recombinant proteins. Binding experiments showed that Mat2A binds tightly to Mat2B with a *K*_d_ of 6 ± 1 nM and a binding stoichiometry of 2:1 (Mat2A/Mat2B) (Figure 6). The steady-state kinetic parameters using conditions where all Mat2A is expected to be in complex with Mat2B were indistinguishable from those of Mat2A alone (Figure 7 and Table 1). We observed a modest, 1.8-fold increase in the potency of SAM inhibition in the presence of Mat2B. Overall, these results suggest that despite Mat2B being a tight binding ligand that stabilizes Mat2A, binding of Mat2B to Mat2A has no significant effect on the catalytic activity of Mat2A or inhibition by SAM to be of physiological relevance. Therefore, if Mat2B expression truly impacts cellular SAM concentrations, it is likely occurring by an unknown mechanism and not by directly regulating the enzymatic activity of Mat2A. Other evidence that supports our *in vitro* data include (1) a comparison of the crystal structure of Mat2A alone with that of the Mat2A/Mat2B complex showing no significant differences in active site residues and (2) kinetic isotope effect experiments that showed no difference in the transition-state structure between Mat2A alone and the Mat2A/Mat2B complex.^13,16,31^ Furthermore, as discussed above, cells possess a mechanism for maintaining SAM homeostasis via the METTL16-mediated methylation of Mat2A mRNA. Presumably, any regulatory effects caused by Mat2B binding would be negated by the action of METTL16.

Structurally, Mat2B closely resembles short-chain dehydrogenase/reductase (SDR) enzymes with a conserved Rossmann fold that bind NADP(H). Mat2B also possesses the conserved SDR catalytic triad (YxxxKS),^40^ suggesting that Mat2B could be a pyridine nucleotide dependent enzyme catalyzing an unknown redox reaction. Overexpression of Mat2B has been demonstrated to increase DNA synthesis in HuH-7 cells, whereas reducing Mat2B levels by RNA interference inhibited DNA synthesis in HepG2 cells.^22^ Identifying the enzymatic function of Mat2B is beyond the scope of this study; however, it is currently being pursued. A second possibility might be to help keep the Mat2A homodimer intact and, therefore, stable and active in the cell as suggested by the stability experiments. Finally, a third possibility is that the role of Mat2B is to localize Mat2A in specific cellular compartments such as the nucleus where the bulk of methylation reactions takes place. This is supported in the literature by reports that Mat2A interacts with a host of proteins (Mat2B, BAF53a, CHD4, and PARP1) to act as a co-repressor complex of MafK, localizing Mat2A in the nucleus and providing SAM for histone methyltransferases.^41,42^ Mat2A activity could be significantly enhanced when it is part of these multiprotein complexes. A cellular compartmentalization function for Mat2B is further supported by reports that the Mat2B protein mostly resides in the nucleus of HepG2 and RKO cells and interacts with a number of nuclear proteins such as DEAD box polypeptide 1 and pre-mRNA cleavage factor I (CFI_m_).^43^ Intriguingly, CFI_m_ is also implicated in regulating SAM homeostasis in concordance with METTL16.^36^ It is hypothesized that METTL16 acts as a SAM sensor that facilitates CFI_m_-mediated splicing of Mat2A mRNA. The differences in SAM concentrations in the cell that have been reported when Mat2B activity is knocked out could therefore be caused by its interaction with CFI_m_, which in turn affects the amount of Mat2A being synthesized via mRNA splicing. Mat2B is described to interact with human antigen R (HuR), an mRNA-binding protein that stabilizes the mRNA of a number of proteins including cyclin D1 and cyclin A, which are required for cell cycle progression.^43^ Mat2B is also reported to form a complex with G-protein-coupled receptor kinase interacting protein-1 (GIT1) affecting the Ras–Raf–MEK signaling pathway.^44^

In summary, Mat2B appears to interact with many nuclear proteins, potentially affecting many different cellular processes. Further investigation is needed to properly understand the cellular regulation of Mat2A, the cellular role of Mat2B, and the potential ramifications that these factors may have on cancer therapies.

## Supporting Information Available

Sequence alignment of Mat2B V1 and Mat2B V2; size-exclusion chromatograms, SDS-PAGE gels, and intact mass spectrums of purified Mat2A and Mat2B; Mat2A concentration dependence MESG assay (assay 1), Michaelis–Menten plot of LC–MS SAM detection (assay 2) ATP-dependence assay, MESG assay (assay 1) representative reaction progress curve upon addition of Mat2A to initiate the reaction, MESG assay phosphate standard curve, the effect of adding inorganic pyrophosphatase to the MESG assay (assay 1), LC–MS SAM standard curve; product inhibition by inorganic pyrophosphate and inorganic phosphate; Mat2A reaction progress curves (assay 1) in the absence and presence of Mat2B; Mat2A activity at a range of Mat2B concentrations (assay 1 and assay 2); Mat2A binding and inhibition by an allosteric inhibitor (compound 28); and Mat2A l-Met dependence LC–MS SAM detection assay (assay 2) after preincubation at 37 °C for 15 min in the absence and presence of Mat2B (PDF)

## Accession Codes

Mat2A, UniProtKB P31153 (METK2_HUMAN); Mat2B, UniProtKB Q9NZL9 (MAT2B_HUMAN); Mat1A, UniProtKB Q00266 (METK1_HUMAN).

## Dedication

This work is dedicated to the memory of John Sears Blanchard.

## Footnotes

1 The specific activity of the native enzyme purified from human lymphocytes is reported to be 12,000 U mg^–1^ protein, where 1 U was defined as 1 nmol SAM product formed h^–1^ at nearly saturating concentrations of ATP and l-Met. The 12,000 nmol mg^–1^ h^–1^ specific activity corresponds to a *k*_cat_ of 0.15 s^–1^ per Mat2A monomer (MW = 43,748 Da).^19^
