# Supplementary material for: Human Mat2A Uses an Ordered Kinetic Mechanism and Is Stabilized but Not Regulated by Mat2B
Source: Biochemistry. 2021 Nov 15;60(47):3621–32. doi: 10.1021/acs.biochem.1c00672 (PMC8638259; doi:10.1021/acs.biochem.1c00672)
Supplement: Supplementary file 1 — bi1c00672_si_001.pdf [file bi1c00672_si_001.pdf]

Human Mat2A uses an ordered kinetic mechanism and is stabilized but not regulated by Mat2B

Jonathan Bailey[1, 2], Holly Douglas[1], Laura Masino[3], Luiz Pedro Sorio de Carvalho[1]*, Argyrides Argyrou[2]*

[1]Mycobacterial Metabolism and Antibiotic Research Laboratory and [3]Structural Biology Scientific Technology Platform, The Francis Crick Institute, London NW1 1AT, United Kingdom. [2]Discovery Sciences, R&D, AstraZeneca, Cambridge CB4 0WG, United Kingdom.

*Corresponding authors

***Luiz Pedro Sorio de Carvalho** – Mycobacterial Metabolism and Antibiotic Research Laboratory, The Francis Crick Institute, London NW1 1AT, United Kingdom; Email: Luiz.Carvalho@crick.ac.uk

***Argyrides Argyrou –** Discovery Sciences, R&D, AstraZeneca, Cambridge CB4 0WG, United Kingdom; orcid.org/0000-0003-3141-9122; Email: argyrides.argyrou@astrazeneca.com

Supporting Information

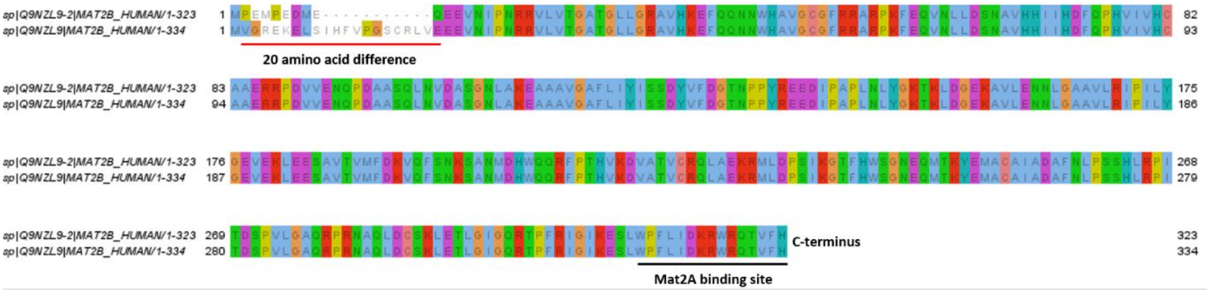

Figure S1. Sequence alignment of Mat2B V1 (bottom) and Mat2B V2 (top). The V1 and V2 sequences are identical apart from the 20 N-terminal residues (underlined in red). The C-terminal Mat2A binding site is underlined in black. Sequences obtained from UniProt and alignment performed using Jalview 2.11.1.4.

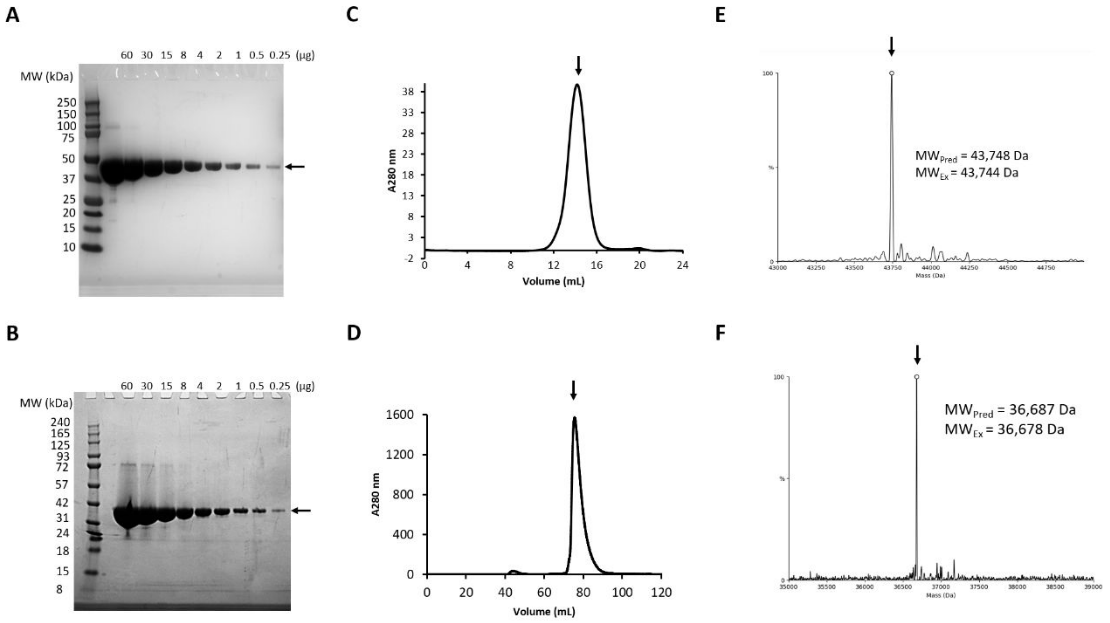

Figure S2. Purification of recombinant human Mat2A and Mat2B by Ni-NTA affinity chromatography and size-exclusion chromatography**.** (A) SDS-PAGE loading series of purified and concentrated Mat2A. (B) SDS-PAGE loading series of purified and concentrated Mat2B. (C) SEC chromatogram of Mat2A**.** (D) SEC chromatogram of Mat2B. (E) Intact mass spectrometry of purified Mat2A. (F) Intact mass spectrometry of purified Mat2B.

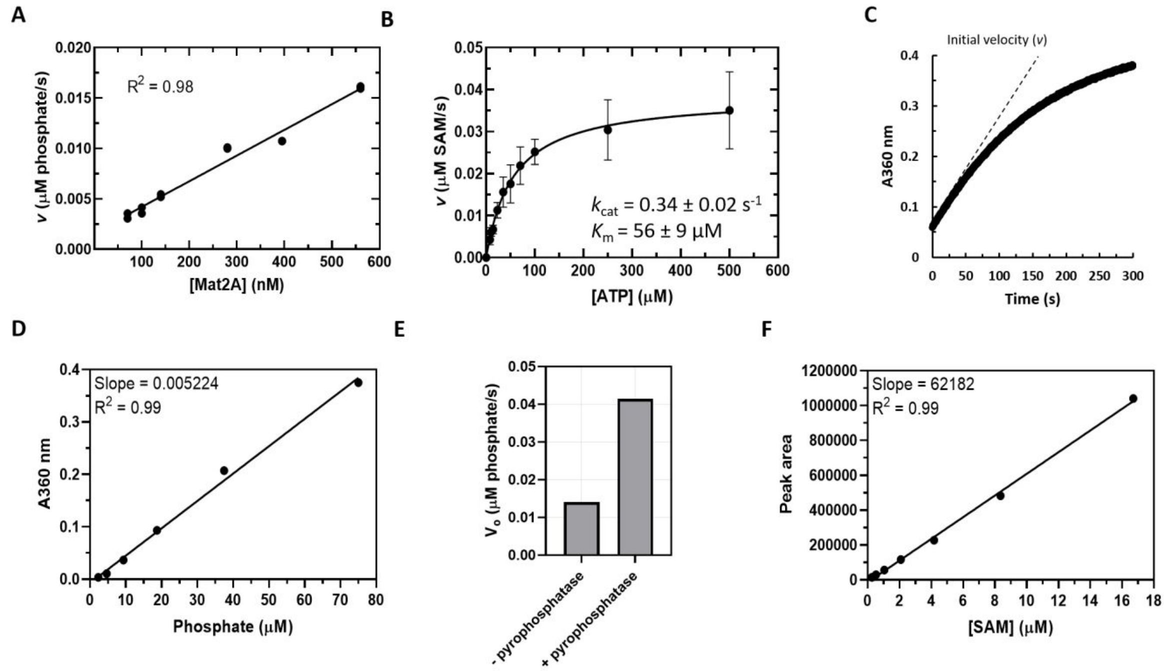

Figure S3. MESG phosphate detection assays and LC-MS SAM detection assays were used to monitor Mat2A activity. (A) Mat2A concentration dependence MESG assay (Assay 1). Mat2A concentrations are 70, 100, 140, 280, 395 and 560 nM. (B) Michaelis-Menten plot of LC-MS SAM detection (Assay 2) ATP-dependence assay. ATP concentrations are 0, 7.8, 10, 14, 23, 35, 50, 70, 100 and 500 µM, L-Met concentration is 600 µM, Mat2A concentration is 115 nM. Plot generated by fitting data to eq 5. (C) . MESG assay representative reaction progress curve upon addition of Mat2A to initiate the reaction. (D) MESG assay phosphate standard curve. (E) EnzChek® Phosphate detection assay (MESG, Assay 1) signal increases 3-fold upon addition of inorganic pyrophosphatase. (F) LC-MS SAM standard curve.

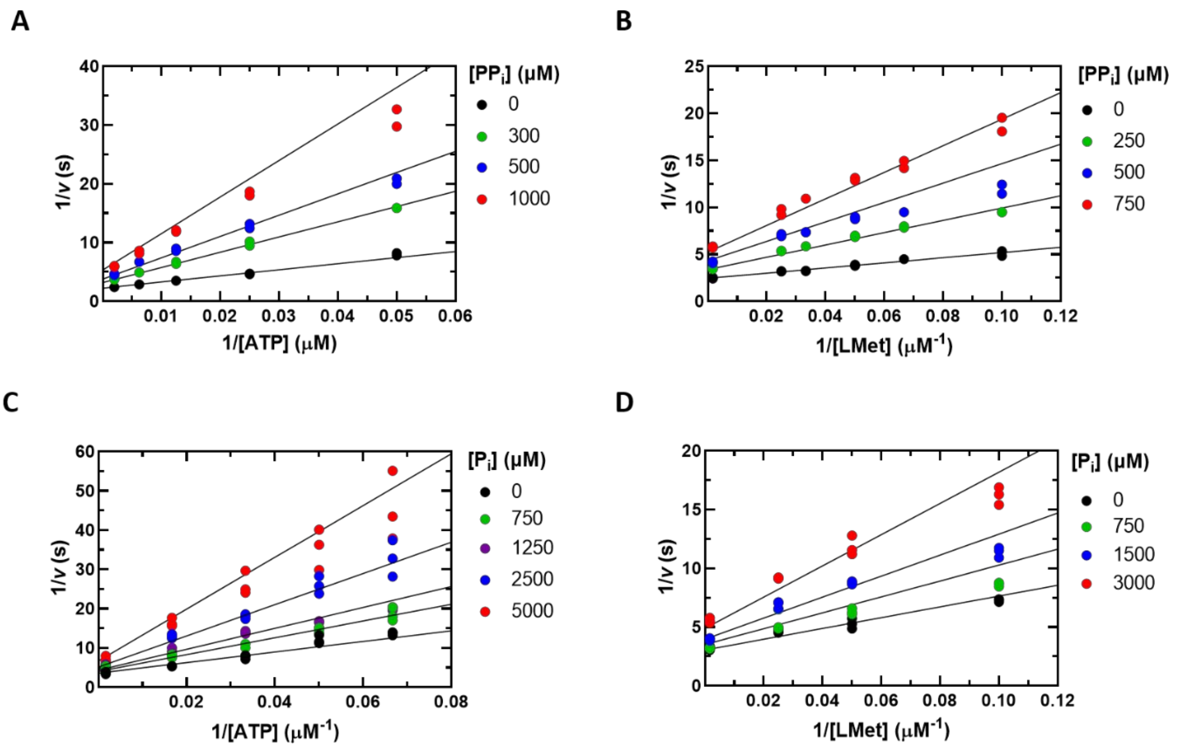

Figure S4. Double reciprocal plots of product inhibition by pyrophosphate (PP_i) and phosphate (P_i) show noncompetitive inhibition. (A) ATP-dependence (20 – 500 µM) pyrophosphate inhibition assay at saturating L-Met (600 µM). (B) L-Met-dependence (10 – 600 µM) pyrophosphate inhibition assay at saturating ATP (500 µM). (C) ATP-dependence (15 – 500 µM) phosphate inhibition assay at saturating (600 µM) L-Met. (D) L-Met-dependence (10 – 600 µM) phosphate inhibition assay at saturating ATP (500 µM). Symbols show experimental data and solid lines show simultaneous fits of all data points to eq 3.

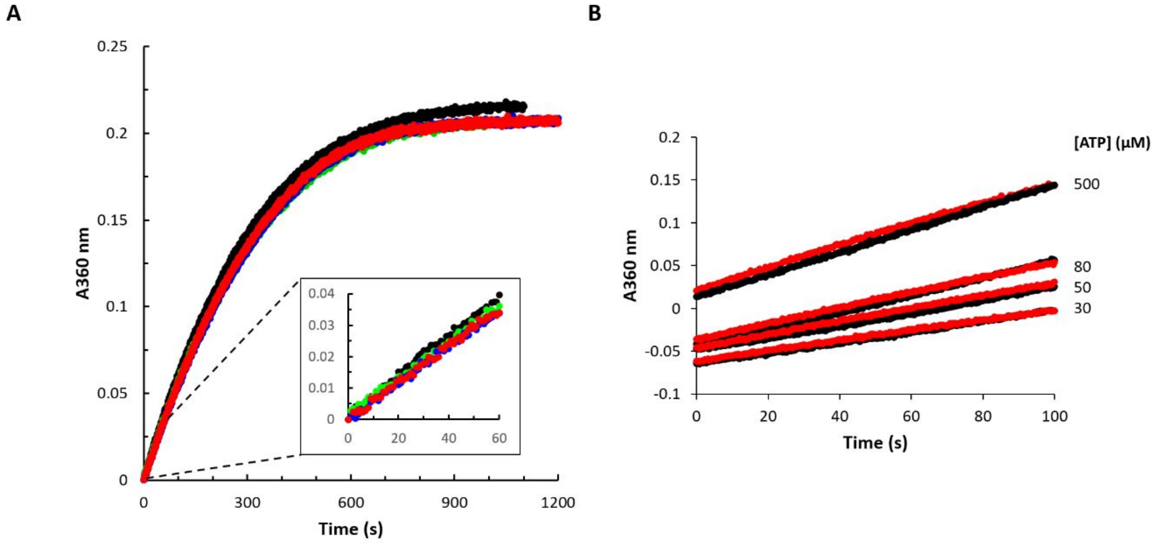

Figure S5. Mat2A reaction progress curves in the absence and presence of Mat2B. (A) Test for slow-onset inhibition. Mat2A concentration is 0.3 µM, L-Met concentration is 10 µM, ATP concentration is 70 µM, Mat2B concentrations are 0 (Black), 0.3 (Green), 3.0 (Blue) and 7.5 (Red) µM. Insert shows the first 60 s of data where rate is linear. (B) ATP concentration-dependence assay. L-Met concentration is 600 µM. Black is 0.25 µM Mat2A only and red is 0.25 µM Mat2A and 0.6 µM Mat2B.

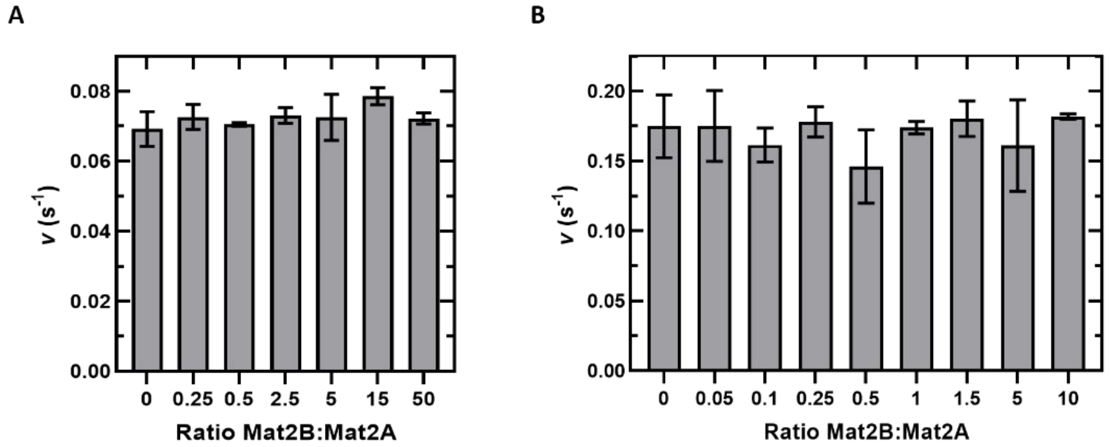

Figure S6. Mat2A activity at a range of Mat2B concentrations. (A) Assay 1 (MESG phosphate detection assay). Mat2A concentration is 0.3 µM, L-Met concentration is 10 µM, ATP concentration is 70 µM, Mat2B concentrations are 0, 0.075, 0.15, 0.75, 1.5, 4.5 and 15 µM. (B) Assay 2 (LC-MS SAM detection assay). Mat2A concentration is 0.3 µM, Mat2B concentrations are 0, 0.015, 0.03 0.075, 0.15, 0.3, 0.45, 1.5 and 3 µM. L-Met concentration is 10 µM, ATP concentration is 1.25 mM.

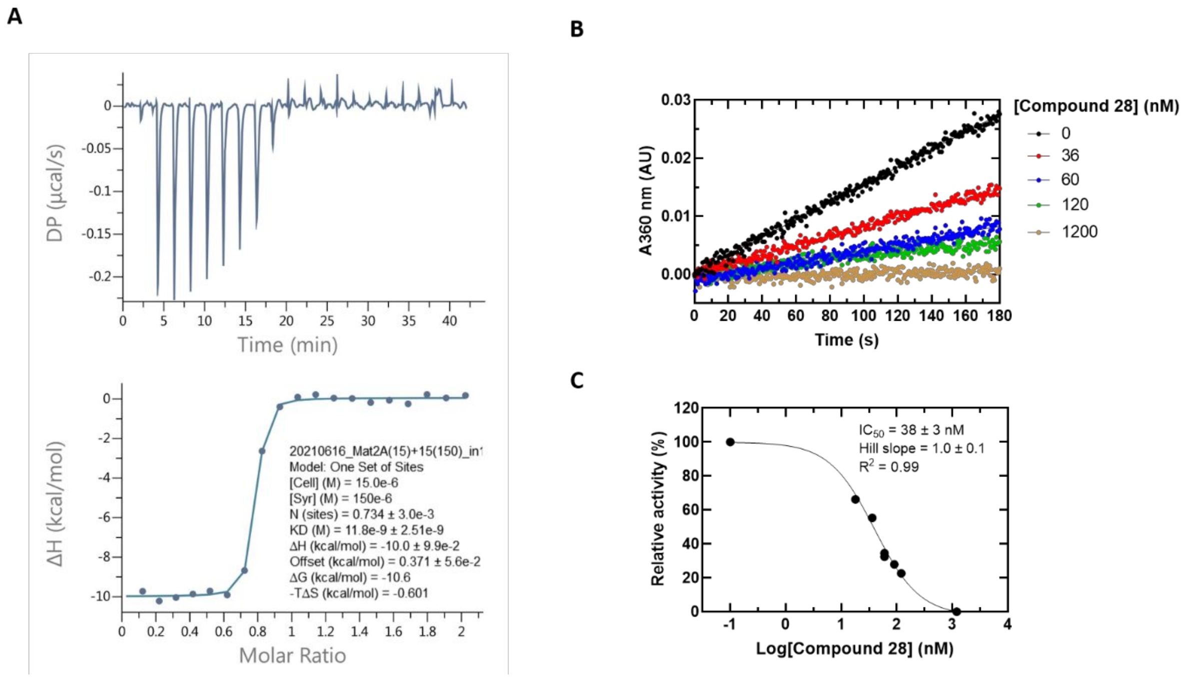

Figure S7. Mat2A binding and inhibition by an allosteric inhibitor, compound 28.[1] (A) ITC thermograms of Mat2A binding to compound 28 (K_d = 12 ± 2.5 nM). Mat2A concentration in the cell is 15 µM, compound 28 concentration in the syringe is 150 µM. Unprocessed thermograms (top panels) and the binding isotherm from the integrated thermogram fit to the One Set of Sites model using the MicroCal PEAQ-ITC analysis software (bottom panels). Circles indicate the integrated heat, and the curve represents the best fit. (B) Reaction progress curves of Mat2A in the presence of varying concentrations of compound 28. Mat2A concentration is 0.06 µM. (C) Concentration-response curve of the rate data shown in (B).

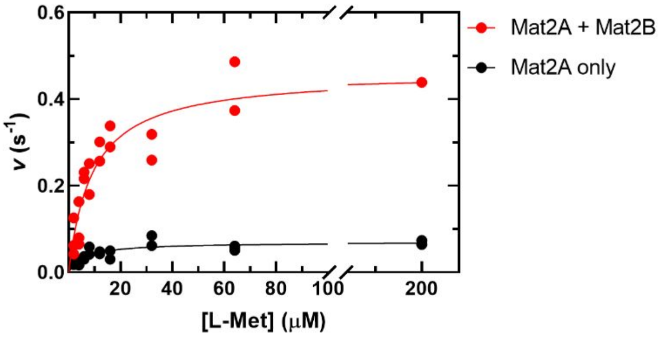

	Mat2A only	Mat2A + Mat2B
$K_{m\ L\text{-}Met}$ (μM)	6.0 ± 1.5	9.0 ± 2.0
k_{cat} (s^{-1})	0.070 ± 0.005	0.46 ± 0.03

Figure S8. Mat2A ± Mat2B L-Met dependence LC-MS SAM detection assay (assay 2) after preincubation at 37 ^{o}C for 15 min. Michaelis-Menten plot and kinetic constants obtained from fitting the initial velocity data to eq 5. L-Met concentrations is varied (2-200 μM) and ATP concentration is fixed at 1.25 mM. Mat2A and Mat2B concentrations during the preincubation are 60 nM and 30 nM, respectively. Mat2A and Mat2B concentrations during the assay are 30 nM and 15 nM, respectively.

References

[1] De Fusco, C., Schimpl, M., Borjesson, U., Cheung, T., Collie, I., Evans, L., Narasimhan, P., Stubbs, C., Vazquez-Chantada, M., Wagner, D. J., Grondine, M., Sanders, M. G., Tentarelli, S., Underwood, E., Argyrou, A., Smith, J. M., Lynch, J. T., Chiarparin, E., Robb, G., Bagal, S. K., and Scott, J. S. (2021) Fragment-Based Design of a Potent MAT2a Inhibitor and in Vivo Evaluation in an MTAP Null Xenograft Model, *J Med Chem 64*, 6814-6826.
